# A side-by-side comparison of different capacitation media in developing mouse sperm fertilizing ability

**DOI:** 10.1038/s41598-024-65134-w

**Published:** 2024-06-21

**Authors:** Lucas N. González, María M. Giaccagli, Jael D. Herzfeld, Patricia S. Cuasnicú, Vanina G. Da Ros, Débora J. Cohen

**Affiliations:** Instituto de Biología y Medicina Experimental (IBYME), Fundación IBYME, Consejo Nacional de Investigaciones Científicas y Técnicas (CONICET), Vuelta de Obligado 2490, C1428ADN Ciudad Autónoma de Buenos Aires, Argentina

**Keywords:** Sperm, Capacitation, Fertilization, Acrosome reaction, Hyperactivation, Cell biology, Developmental biology

## Abstract

To acquire the ability to fertilize the egg, mammalian spermatozoa must undergo a series of changes occurring within the highly synchronized and specialized environment of the female reproductive tract, collectively known as capacitation. In an attempt to replicate this process in vitro, various culture media for mouse sperm were formulated over the past decades, sharing a similar overall composition but differing mainly in ion concentrations and metabolic substrates. The widespread use of the different media to study the mechanisms of capacitation might hinder a comprehensive understanding of this process, as the medium could become a confounding variable in the analysis. In this context, the present side-by-side study compares the influence of four commonly used culture media (FD, HTF and two TYH versions) on mouse sperm capacitation. We evaluated the induction of protein kinase A phosphorylation pathway, motility, hyperactivation and acrosome reaction. Additionally, in vitro fertilization and embryo development were also assessed. By analyzing these outcomes in two mouse colonies with different reproductive performance, our study provides critical insights to improve the global understanding of sperm function. The results obtained highlight the importance of considering variations in medium composition, and their potential implications for the future interpretation of results.

## Introduction

Sperm capacitation, transport of gametes, fertilization and early embryo development are physiological events that occur in a highly synchronized manner within the female reproductive tract, which provides the appropriate environment for their progression. In particular, capacitation involves a series of functional and structural changes critical for sperm to acquire the ability to fertilize the egg^1,2^. Two functional endpoints of capacitation are the acrosome reaction (AR), a unique exocytic event in the sperm head, and hyperactivation (HA), a vigorous flagellar movement, both required for sperm penetration of the egg vestments and gamete fusion^3^.

Sperm manipulation in vitro provides a powerful and essential tool not only for understanding gamete physiology, but also for clinical applications and domestic animal reproduction. After the pioneering work by Toyoda and co-workers^4^, who first reported the successful in vitro fertilization (IVF) of mouse eggs using a “chemically defined medium”, TYH for Toyoda, Yokoyama and Hosi, a great bulk of research has been carried out to underpin the mechanisms involved in capacitation (reviewed in Ref^5,6^). To allow this process in vitro, defined medium compositions attempt to reflect the conditions to which spermatozoa are exposed in the female reproductive tract^7^. However, the fact that uterine and oviductal fluids exhibit temporal and spatial variations in their composition along the tract, influenced by the timing of the ovulatory cycle^8,9^, challenged in vitro replication. As a consequence, different mouse sperm capacitation media were described in the literature, based on the Krebs–Ringer bicarbonate medium (i.e., TYH, CZB, or Whittens) or on modified Tyrode’s solution (i.e., T6, or FD named after the developers Fraser and Drury^10^). In addition, a Human Tubal Medium (HTF), adapted for the mouse model by Nakagata^11^, is also broadly used. While the majority of them exhibit a similar formula, distinctions arise primarily in terms of ion concentrations and metabolic substrates, opening the possibility that the development of sperm capacitation differs depending on the medium used. Moreover, these media are employed indiscriminately in different laboratories worldwide to assess capacitation, often without due consideration for the potential effects that the variations in their compositions could exert on this process. This situation becomes even more complex considering that: (1) the choice of the medium fluctuates over the decades and can also be influenced by its commercial availability; (2) the variations in the medium composition from the original recipes are not always included in publications; and, finally (3) different mouse strains with distinct reproductive performances are used for these studies. All these factors can lead to overestimation or underestimation of the obtained results across the literature, confounding the drawn conclusions.

In view of this, in the present study, we simultaneously analyzed the extent to which four different capacitation media influence the development of mouse sperm fertilizing ability. The chosen media are the most commonly used in the literature and represent the various previously mentioned basic formulations. For evaluating capacitation, we analyzed early events such as the protein kinase A (PKA) signaling pathway, and late events such as HA and AR. Additionally, IVF and embryo development assays were also performed to get a better comprehension of the functional phenomena that occur during capacitation, since only fully capacitated sperm are capable of fertilizing the egg and supporting early development^3,12^. Taking into consideration the different reproductive performances among mouse strains, two colonies were included in the study: C57BL/6 inbred strain with low performance^13^ and B6CF1 hybrids with high performance^14^. To our knowledge, the systematic comparison carried out herein offers a critical perspective that could improve the interpretation of the results obtained worldwide. In addition, our study provides insights that could contribute to a deeper understanding of how different factors influence sperm function.

## Results

### Sperm functional parameters in different capacitation media

For this study, we selected the four most commonly used mouse sperm capacitation media: FD, HTF, TYH and TYH (HEPES), whose composition is listed in Table 1.

**Table 1:** Composition of the capacitation media.

Component	FD^10^	HTF^11^	TYH^4^	TYH (HEPES)^4,12^
NaCl (mM)	99.3	101.6	119.4	119.4
KCl (mM)	2.7	4.7	4.7	4.7
CaCl_2_ (mM)	1.8	5.1	1.7	1.7
MgCl_2_ (mM)	0.5	–	–	–
MgSO_4_ (mM)	–	0.2	1.2	1.2
KH_2_PO_4_ (mM)	–	0.4	1.2	1.2
Na_2_HPO_4_ (mM)	0.3	–	–	–
NaHCO_3_ (mM)	25	25	25	15
HEPES (mM)	–	–	–	20
Glucose (mM)	5.6	2.8	5.6	5.6
Na-lactate (mM)	24.4	21.4	–	–
Na-pyruvate (mM)	0.5	0.3	0.5	0.5
BSA (mg/ml)	3	4	4	5

**Figure 1:**
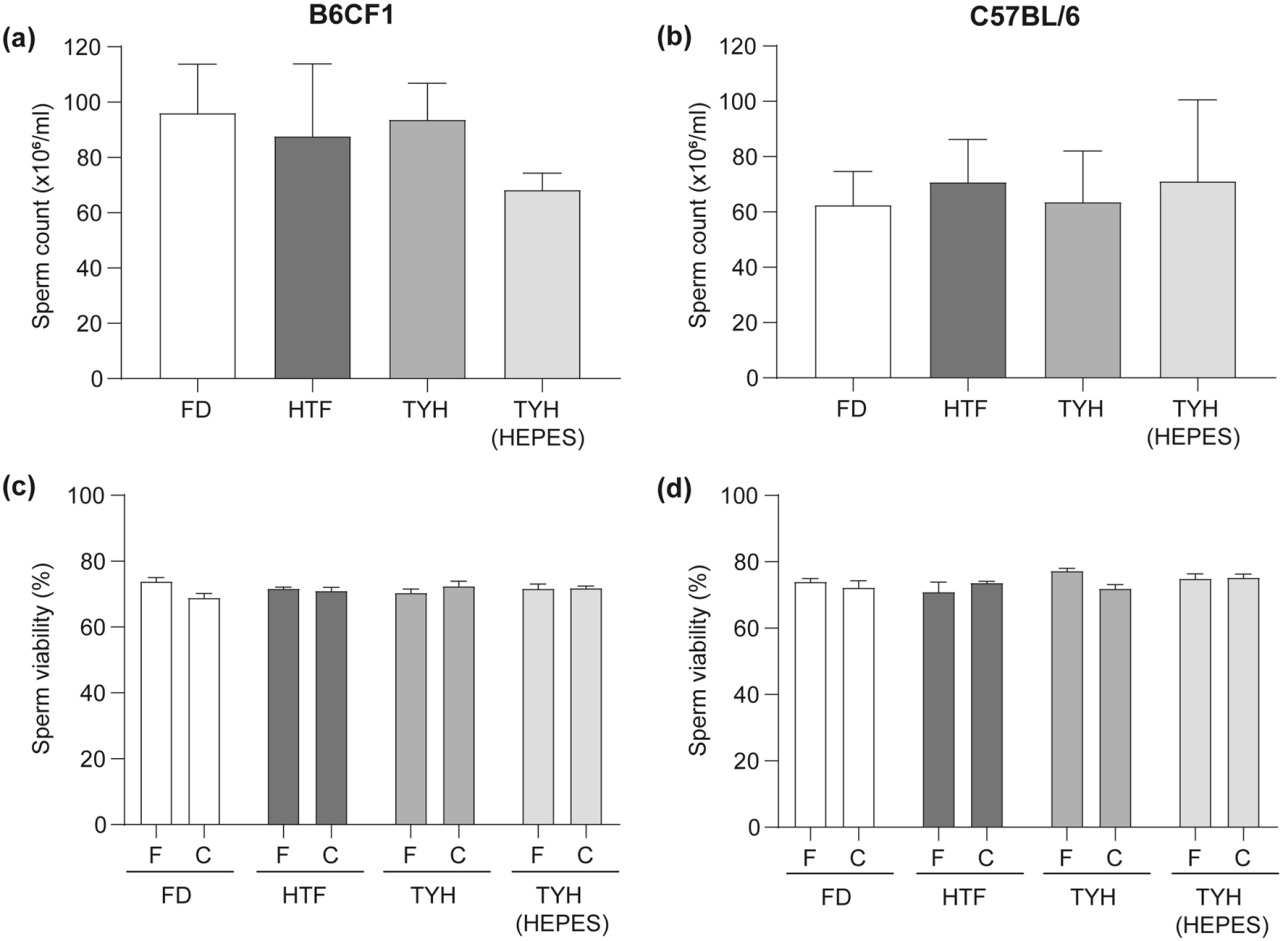
Sperm count and viability in the different media. Sperm from B6CF1 (**a**, **c**) and C57BL/6 (**b**, **d**) animals were recovered by “swim-out” (F, fresh) and then capacitated in the corresponding media for 90 min (C, capacitated sperm). Sperm count (**a**, **b**) was recorded immediately after “swim-out” in each medium. Viability of fresh and capacitated sperm (**c**, **d**) was determined by eosin staining. Data are presented as mean ± SEM of 3 independent experiments. No significant differences were observed among groups.

As a first step, we analyzed the number of spermatozoa recovered after “swim-out” in each medium as well as the percentage of viable sperm before and after capacitation. Similar results were obtained in all conditions (Fig. 1). Subsequently, we simultaneously compared in the different media the level of induction of several sperm parameters occurring during capacitation, such as phosphorylation of PKA substrates (pPKAs), motility, HA and AR.

**Figure 2:**
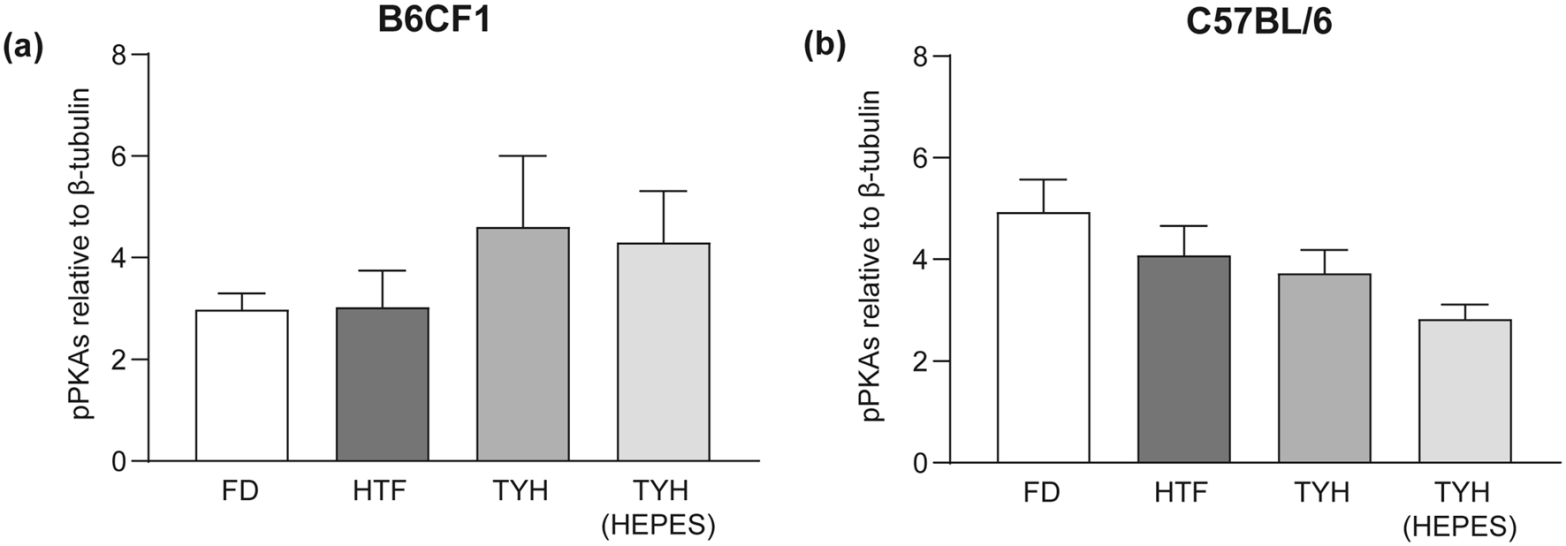
Phosphorylation of PKA substrates (pPKAs) in the different media. Sperm from B6CF1 (**a**) and C57BL/6 (**b**) animals were capacitated in the corresponding media for 90 min. Levels of pPKAs were analyzed by Western blotting. Pixel intensity for each lane was quantified and relativized to that of the β-tubulin band (loading control). Data are presented as mean ± SEM of at least 4 independent experiments. No significant differences were observed among groups.

To evaluate PKA activation, one of the first signaling events observed during sperm capacitation^15^, pPKAs levels in the different media were analyzed by Western blotting. As expected, the typical pattern of phosphorylation was induced in all cases after capacitation (Suppl. Fig. 1). Moreover, results showed that the quantification of the relative abundance of pPKAs exhibited no differences among media for both B6CF1 and C57BL/6 sperm (Fig. 2a and b).

**Figure 3:**
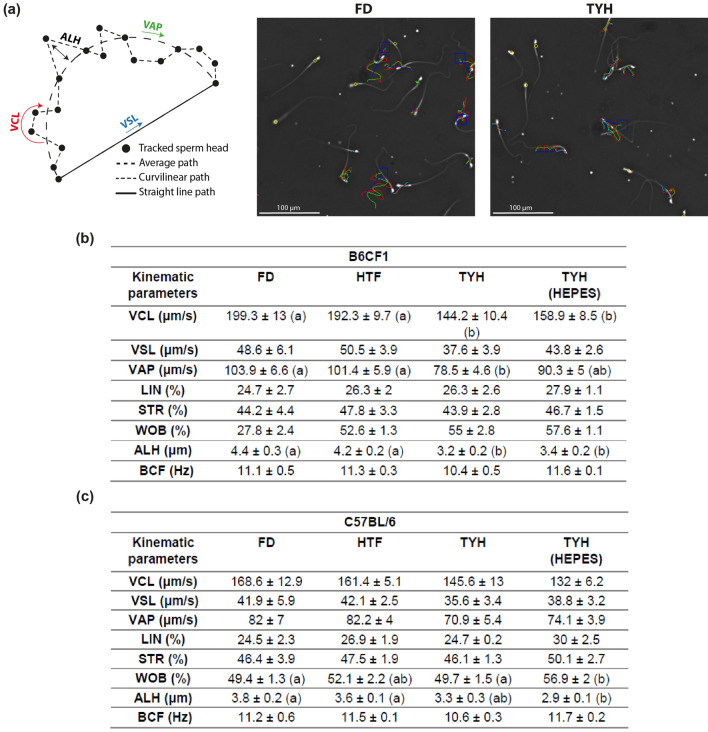
Motility parameters assessed by Computer-Assisted Sperm Analysis (CASA) in the different media. Epididymal sperm were incubated in different capacitation media for 90 min and kinematic parameters were measured by CASA. (**a**) Schematic representation of the measured parameters (left panel) and examples of microscope fields used for quantification of B6CF1 sperm capacitated in FD (central panel) or TYH (right panel) medium. For each cell, VCL is shown in red, VSL, in blue and VAP, in green. Blue squares depict hyperactivated sperm whereas yellow circles, immotile sperm. (**b**, **c**) Quantification of all the kinematic parameters measured in B6CF1 (**b**) and C57BL/6 (**c**) sperm capacitated in the corresponding media. VCL, curvilinear velocity; VSL, straight line velocity; VAP, average path velocity; LIN, linearity (VSL/VCL × 100); STR, straightness (VSL/ VAP × 100); WOB, oscillation index (VAP/VCL × 100); ALH, amplitude of lateral head; BCF, beat frequency. n = 5. Statistically significant differences among groups are denoted with distinct letters, *p* < 0.05.

**Figure 4:**
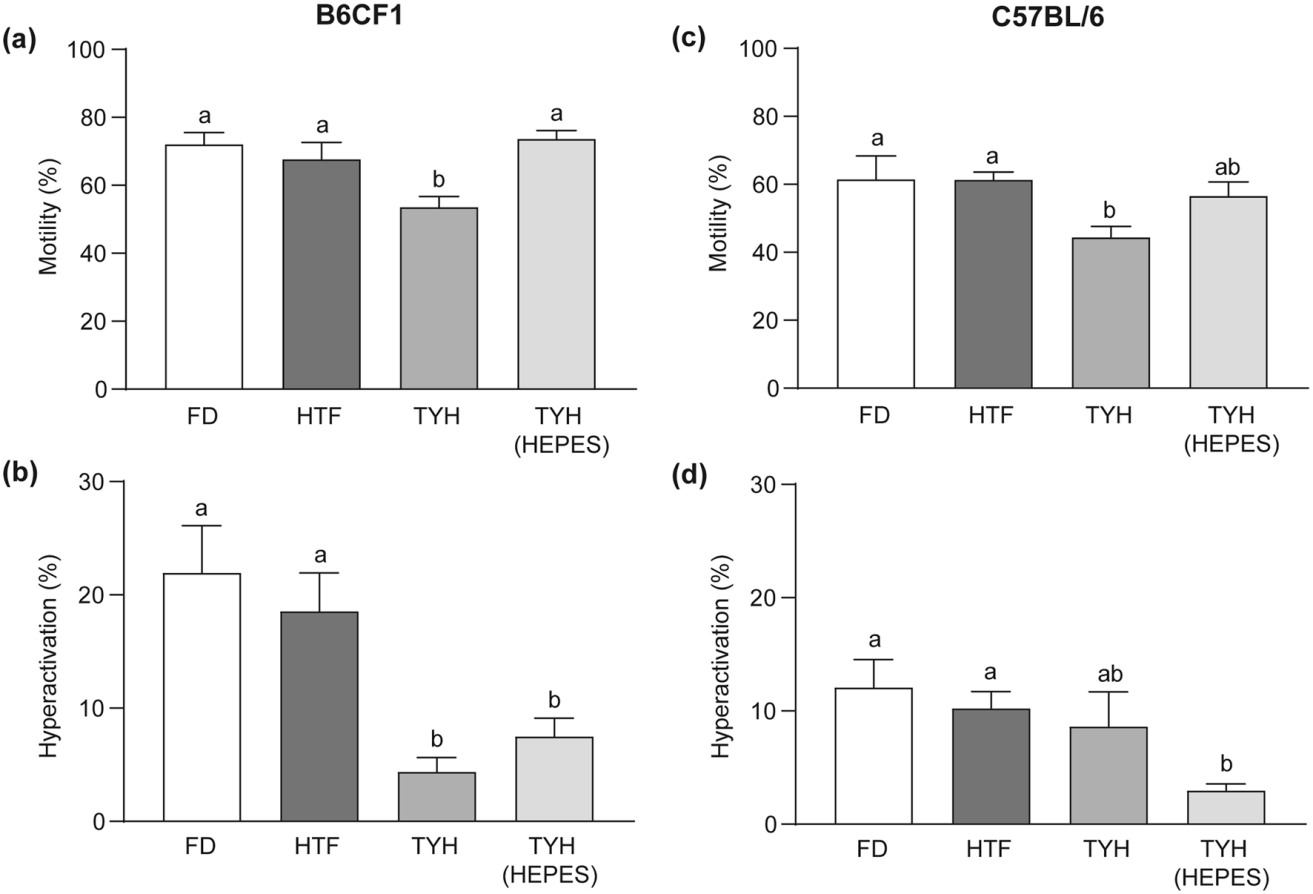
Motility parameters in the different media. Sperm from B6CF1 (**a**, **b**) and C57BL/6 (**c**, **d**) animals were capacitated in the corresponding media for 90 min. Percentage of total motility (**a**, **c**) and hyperactivation in motile sperm (**b**, **d**) were determined by CASA. Data are presented as mean ± SEM of 5 independent experiments. Bars with distinct letters are significantly different, *p* < 0.05.

Next, an objective analysis of sperm motility using Computer-Assisted Sperm Analysis (CASA) was performed after capacitation in the different culture media (Fig. 3a). In B6CF1 samples, total motility was lower in TYH, in contrast to the other tested media (Fig. 4a). Furthermore, in TYH and TYH (HEPES) several kinematic parameters (Fig. 3b) and the percentage of hyperactivated sperm (Fig. 4b) were significantly lower compared to the remaining media. A similar situation was observed in C57BL/6 samples, in which total motility was also lower in TYH in contrast to FD and HTF whereas values were intermediate in TYH (HEPES) (Fig. 4c). On the other hand, two kinematic parameters (WOB and ALH, Fig. 3c) and the percentage of hyperactivated sperm (Fig. 4d) were significantly lower in TYH (HEPES) compared to FD and HTF. In this case, TYH did not produce significant differences in these parameters relative to the other culture media.

**Figure 5:**
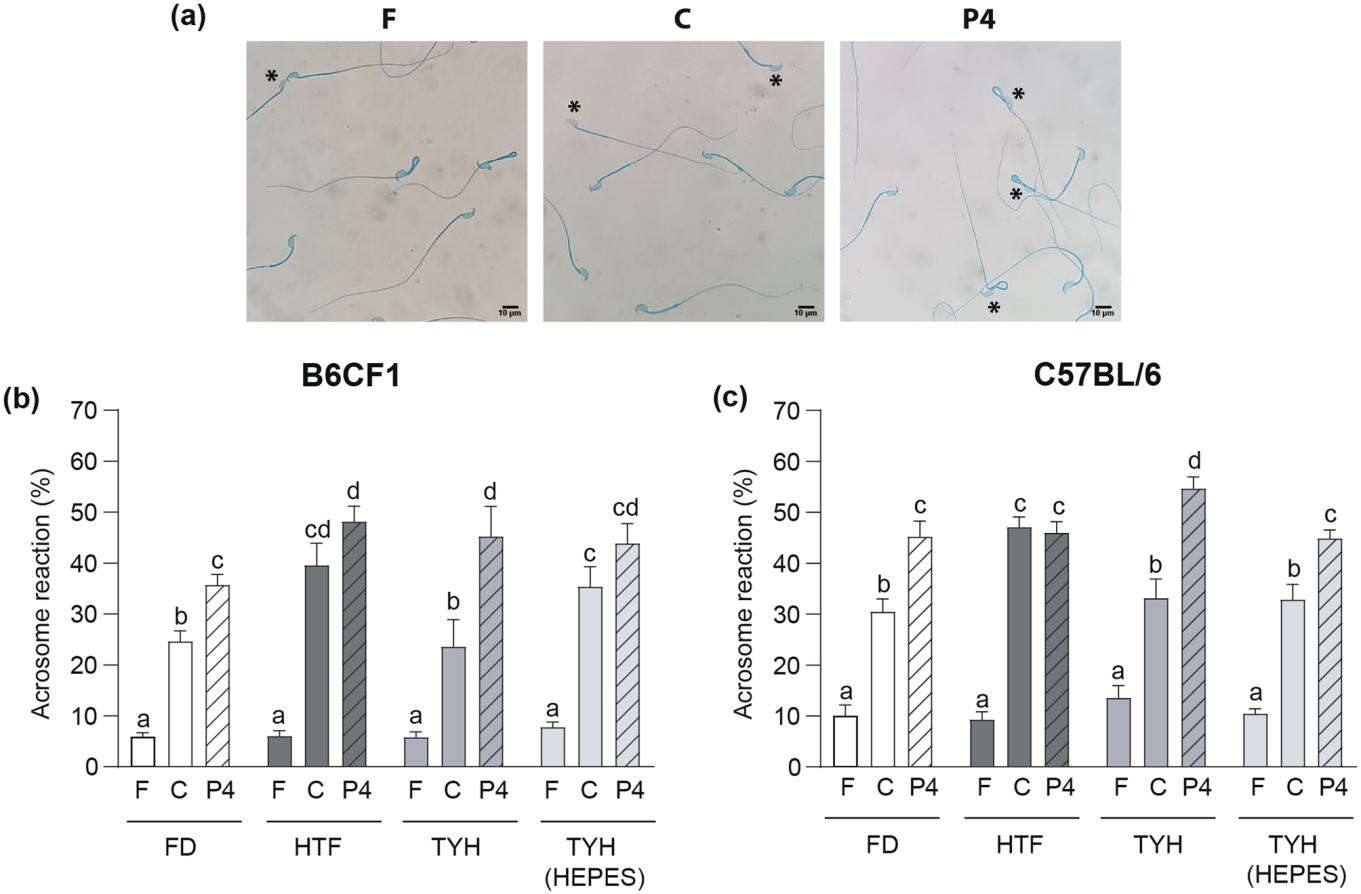
Acrosome reaction (AR) in the different media. (**a**) Examples of representative microscope fields of AR determined by Coomassie Brilliant Blue staining in fresh (F), capacitated (C) and progesterone-treated (P4) C57BL/6 sperm incubated in FD. Asterisks denote acrosome-reacted sperm. Sperm from B6CF1 (**b**) and C57BL/6 (**c**) animals were capacitated in the corresponding media for 90 min. Percentage of AR was determined in fresh, capacitated and progesterone-treated sperm as in (**a**). Data are presented as mean ± SEM of at least 5 independent experiments. Bars with distinct letters are significantly different, *p* < 0.05.

The occurrence of AR was also evaluated in the different media by Coomassie Brilliant Blue staining. As expected, a significant increase in spontaneous AR was observed after sperm capacitation in all media and for both animal colonies (Fig. 5a). Furthermore, the physiological AR-stimulant progesterone (P4) induced AR in all tested conditions, except in HTF, in either B6CF1 or C57BL/6 (Fig. 5b and c).

When comparing the levels of AR induction produced by each medium after capacitation in B6CF1 sperm (Fig. 5b), it was observed that while the spontaneous AR was higher with HTF and TYH (HEPES), the P4-induced AR was lower with FD (Fig. 5b). In the case of C57BL/6 sperm (Fig. 5c), the highest level of spontaneous AR was also recorded in HTF, while TYH supported the highest value of P4-induced AR.

### Sperm fertilizing ability in different capacitation media

To functionally analyze the impact of capacitating sperm in the different media, IVF assays were carried out. These studies were performed with cumulus-oocyte complexes (COC), zona pellucida (ZP)-intact and ZP-free eggs as sperm require different functional characteristics derived from capacitation to traverse each of the layers surrounding the egg and to fuse with the egg membrane. Given the possibility that sperm complete capacitation in the fertilization droplet^16^, gamete co-incubations were conducted using the same medium employed for capacitation.

**Figure 6:**
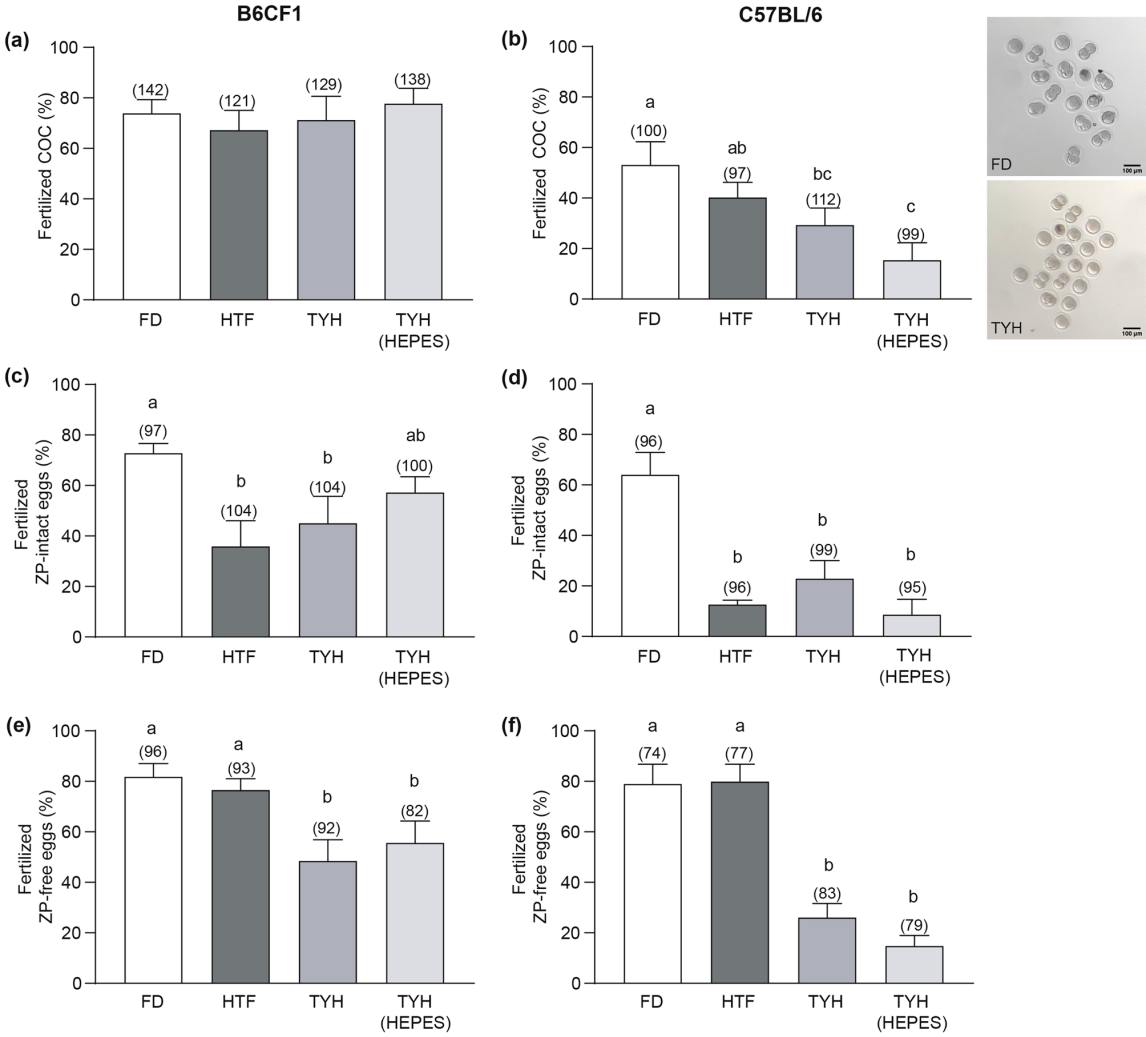
In vitro sperm fertilizing ability in the different media. Sperm from B6CF1 (**a**, **c**, **e**) and C57BL/6 (**b**, **d**, **f**) animals capacitated in the corresponding media were used to inseminate COC (**a**, **b**), ZP-intact (**c**, **d**) and ZP-free eggs (**e**, **f**). In (**a**) to (**d**), the percentage of 2-cell embryos was determined. The inset in (**b**) corresponds to representative biological images of fertilization experiments with COC inseminated with C57BL/6 sperm capacitated in FD (upper panel) or TYH medium (lower panel). In (**e**) and (**f**), the percentage of eggs with at least one decondensing sperm nucleus in the cytoplasm was determined by Hoechst 33342 staining. Data are presented as mean ± SEM of at least 5 independent experiments. In brackets is indicated the total number of analyzed eggs per treatment. Bars with distinct letters are significantly different, *p* < 0.05.

When COC were inseminated, similar fertilization rates, measured as the percentage of 2-cell embryos, were obtained for B6CF1 sperm capacitated in the different media (Fig. 6a). In contrast, for C57BL/6 sperm, incubation in FD and HTF produced higher fertilization rates than in TYH (HEPES), while TYH supported intermediate levels (Fig. 6b).

In the case of cumulus-free ZP-intact eggs, when B6CF1 gametes were used (Fig. 6c), incubation in FD resulted in higher fertilization rates compared to those obtained in HTF and TYH, while TYH (HEPES) produced intermediate results. Regarding gametes from C57BL/6 mice (Fig. 6d), the highest fertilization rates were obtained when FD was used.

Finally, gamete fusion was evaluated by incubating capacitated sperm with ZP-free eggs, and analyzing the presence of at least one decondensing sperm nucleus in the egg cytoplasm by Hoechst staining. Results showed that FD and HTF supported the highest fertilization rates for sperm from both B6CF1 (Fig. 6e) and C57BL/6 (Fig. 6f) mice. Polyspermy was not observed in any case (data not shown).

Considering that early embryo development might be affected by differential conditions during sperm capacitation^12^, development to the blastocyst stage was also analyzed. For this, 2-cell embryos, obtained from IVF with COC and sperm capacitated in the different media, were incubated in KSOM (Potassium Simplex Optimized Medium), and the percentages of blastocysts were recorded. Results showed that these rates were not affected by the capacitation media used, regardless of the animal background (Table 2).

**Table 2:** Effect of sperm capacitation in the different culture media on in vitro early embryo development.

Mice	Embryos	FD	HTF	TYH	TYH (HEPES)
B6CF1	Embryo development (%)	90.1 ± 3	96.2 ± 2.6	80.8 ± 5.4	82.2 ± 6.7
	Experiments (No)	7	7	7	7
	Total 2-cell embryos (No)	109	82	91	110
	Total blastocyst (No)	100	78	77	92
C57BL/6	Embryo development (%)	53.3 ± 15.1	55.6 ± 11.9	40.7 ± 17.2	50 ± 20.4
	Experiments (No)	5	5	4	5
	Total 2-cell embryos (No)	50	42	32	15
	Total blastocyst (No)	29	22	15	8

## Discussion

Disparity in the reported levels of in vitro mouse sperm performance observed when using indistinctly different capacitation media and experimental conditions (Ref^12,17–22^, among others), led us to carry out the current side-by-side medium comparison. Our study exposes the differential competence of media in sustaining sperm capacitation in a single specific experimental condition in two mouse models with different reproductive success.

Initial results showing that sperm count and viability were comparable after recovery in each medium set the stage for the following studies. Of note, higher sperm numbers with similar viability were obtained in the hybrid colony with high reproductive performance compared with the inbred C57BL/6. As an early event in capacitation, protein phosphorylation mediated by PKA was evaluated, finding similar levels of induction among the various media and mouse colonies employed. This is in line with the fact that all the tested media contain HCO_3_^−^ concentrations similar to those reported to produce maximum accumulation of cAMP^23^, required for PKA activation. Therefore, as PKA activation is a pivotal step that triggers a variety of molecular and cellular events of capacitation (reviewed in Ref^24^), its robustness is plausible. Considering the well-established relevance of cAMP for the development of late events of capacitation (reviewed in Ref^25,26^), the above results could suggest that AR and HA levels would have been consistent among the different media. However, the fact that our results showed otherwise implies that the specific signaling pathways underlying HA and AR inductions, whether downstream or alternatives to cAMP, depended on media and animal colonies used. Interestingly, we observed a higher degree of susceptibility for HA development than for AR. Indeed, both events required distinct cAMP downstream pathways: i.e. cAMP-induced PKA signaling pathway for HA, and alternative cAMP targets such as EPAC for AR^27–30^.

Upon detailed analysis of each capacitation endpoint, we observed that total sperm motility and HA reached levels comparable to those separately reported in the literature^12,19,21,31–33^. Interestingly, our synchronized comparison of the four media revealed that FD and HTF were the most suitable for boosting both types of motility in the present experimental conditions. This enhancement can be explained by the lactate included in FD and HTF but not in TYH formulas. However, recent evidence has not shown significant differences in these parameters irrespective of whether this substrate was added to the medium or not^33^. On the other hand, although Ca^2+^ is one of the most important factors for HA development^34^, the higher Ca^2+^ concentration of HTF compared to FD did not modify HA levels. In addition, although we observed an intermediate situation in TYH in terms of inducing HA in C57BL/6 mice, the presence of HEPES proved unfavorable for this specialized motility in both colonies. Thus, caution should be taken when comparing HA values across the literature between TYH-based and non TYH-based media. Moreover, consistent with the reproductive success of each colony, the doubling of the HA levels observed in B6CF1 sperm compared to C57BL/6 introduces an additional layer of complexity to the comprehensive interpretation of motility results reported in the bibliography.

Regarding AR induction, although all media successfully promoted spontaneous AR after capacitation at rates comparable to the literature^18,35–37^, discrepancies were observed when sperm were incubated with P4. In this context, TYH produced the highest ratio between P4-induced and spontaneous AR. While the reasons for this advantageous effect remain unclear, we could speculate that the interplay between the concentration of crucial ions, such as Ca^2+^ and K^+^, might contribute to finely tuning the AR response in the different media. In this sense, the fact that HTF sustained the highest spontaneous AR levels compared to the other media could be associated with its elevated Ca^2+^ concentration, given the well-established relationship between AR and extracellular Ca^2+^ concentration^38,39^. Moreover, AR could have achieved a physiological maximum response in HTF that precluded the expected P4 induction. Overall with HA results, the Ca^2+^ levels in HTF appear to differentially impact AR and HA, suggesting distinct susceptibility to Ca^2+^ for each phenomenon. It is interesting to note that the different media showed similar competence to induce AR in both colonies, unlike that observed for HA induction. This reinforces once again that signaling pathways leading to HA and AR present different sensitivity to the in vitro incubation conditions and reproductive performance of the colonies studied here.

As successful fertilization unequivocally signifies that the fertilizing spermatozoon completed capacitation, we next analyzed the extent to which the different capacitation media sustained the development of the sperm fertilizing ability. In this sense, whereas the sperm functional requirements for cumulus penetration are still unclear, AR and HA are essential for ZP-penetration, and AR is necessary for gamete fusion^6^. Therefore, the use of eggs with or without coats in our side-by-side IVF study enabled us to raise conclusions of functional aspects of the capacitation process. Regarding FD, our results support a high performance for this medium to sustain sperm fertilizing ability in the three different stages of the fertilization process and for the two different mouse colonies in the experimental conditions employed here. This is consistent with the levels of AR and HA achieved in this medium.

When using HTF we observed high levels of both IVF with COC and gamete fusion, yet encountering impairments in the ZP penetration stage. These results suggest that sperm capacitated in HTF are not adequately primed for passing through the ZP. The fact that this medium sustained high HA and AR levels opens the possibility that HTF could not support other capacitation and/or gamete co-incubation-associated events not evaluated here and relevant for ZP penetration. Interestingly, in the context of IVF with COC this limitation was overcome, confirming the known beneficial effect of cumulus cells on fertilization^6,40^.

In the case of TYH-based media, ZP penetration and gamete fusion stages were not favored by these experimental conditions. Considering that these two steps are sequential, the results suggest that sperm capacitated in these media exhibited impaired fusion ability. Taking into account the high levels of AR detected in TYH, the observed impairment would not rely on the exocytic event per se but possibly on other critical changes consequent of AR not evaluated by Coomassie Blue staining, such as the acquisition of the fusogenicity of the equatorial segment^3^. Moreover, TYH-capacitated sperm may additionally present limitations in their ability to penetrate the ZP, consistent with the low HA measured by CASA. Concerning IVF with COC, a more complex scenario was observed, depending on the colony used: (i) Sperm from B6CF1 mice exhibited high fertilization rates. Although these findings appear to contradict those previously observed with ZP-intact and ZP-free eggs, they could be explained in the context of the previously mentioned positive effect that cumulus cells have on fertilization. In this case, the presence of the cumulus cells may have bypassed the medium limitations, enabling sperm to complete capacitation within the cumulus layer and to consequently develop HA (required for ZP penetration) and acquire fusion ability. Moreover, these sperm samples were also able to pass through the cumulus layer. Regardless of whether the fully capacitated state acquired within the cumulus cells may also have facilitated penetration of this layer, the need of HA for this stage is still not fully proved. This debate is hindered by the existence of different mouse models in which sperm, despite showing deficient HA, are able to penetrate the cumulus, while others are not^19,41–45^. In view of this, our findings add evidence to support that this analysis should be done considering the different challenges faced by sperm that have such deficiency but caused by different molecular bases (i.e. lack of egg-ligand proteins^42,43^, epididymal maturation deficiencies^41^, metabolic impairment^19^, incomplete capacitation, in this study). (ii) Sperm from C57BL/6 showed the lowest fertilization percentage in TYH-based media. In this case, the effect of cumulus cells seems not to be enough to compensate for incubation conditions, as these sperm might need more stimuli than hybrid ones to become capable of fertilizing COC.

Finally, considering that the study of early embryonic development to the blastocyst stage with the different capacitation media showed results similar to those reported^12,46,47^ in all conditions tested, the choice of a medium for producing a high number of blastocysts relies on the IVF results. Therefore, our results indicate that whereas all the media are suitable for the B6CF1 animals, FD and HTF are preferable media to get the highest blastocyst numbers in C57BL/6 animals. The beneficial factors of these media, including the relevance of each carbon source, are yet to be determined. Furthermore, the comparison between the two mouse colonies revealed that B6CF1 animals consistently demonstrated higher embryo production rates than C57BL/6 mice. Although expected, the underlying mechanisms (i.e. DNA fragmentation, genetic and epigenetic landscapes) for this difference require further studies. The fact that higher embryo production rates are sustained in each of the tested capacitation media suggests that sperm from individuals from a colony with a superior reproductive outcome, such as hybrid animals and outbred strains, possess a more solid ability to adapt to diverse microenvironmental conditions compared to those with a lower reproductive performance (homogeneous inbred strains).

**Figure 7:**
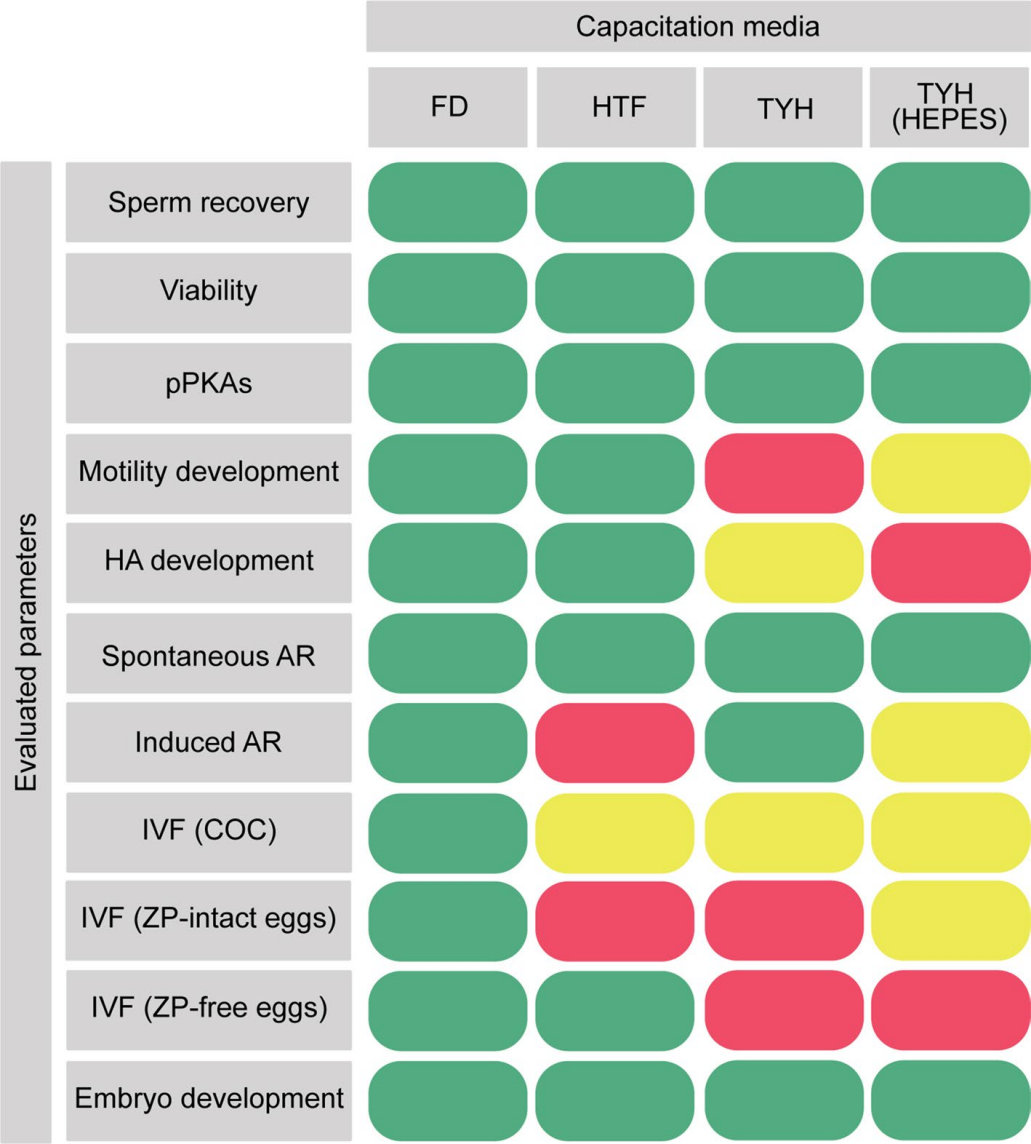
Side-by-side comparison of the outcomes of sperm capacitated in the different media. Colors were assigned as follows: Green: medium supports the specific sperm parameter in both mouse colonies; Yellow: medium differently supports the specific sperm parameter in each mouse colony; Red: medium partially supports the specific sperm parameter in both mouse colonies.

In conclusion, the joint analysis of variations in fertilization success observed along this study corroborates that both the in vitro sperm capacitation conditions and the reproductive performance of the mouse colony are determining factors for experimental designs aimed at understanding the mechanisms underlying gamete physiology. In the context of these mechanisms, our results reveal that certain capacitation events remain robust in the face of fluctuations introduced by media and mouse genetic backgrounds, while others are more dependent on these factors (Fig. 7). In this sense, those variable events can be improved with different experimental conditions. For example, the poor fertilization of BALB/c strain in TYH has been previously addressed by sequential incubation in two different media^48^. Others reported better fertilization success in TYH when increasing the medium volume during gamete co-incubation^16^ or the incubation time, allowing sperm a longer period to capacitate and fertilize the egg^21^. Despite the fact that this high sensitivity to the culture medium conditions can be compensated, it is important to bear in mind the implications that the medium can have for a specific step of sperm capacitation. In this regard, our study may have a potential limitation since we tested a singular experimental condition. While we cannot rule out the possibility that results may differ under other conditions, the observed variability across media would likely persist. Therefore, to mitigate the impact of these variations, we suggest that future manuscripts include more detailed information on these experimental aspects, which would be beneficial for the field. Finally, the implications of this work transcend, to some extent, the particular results we have described and open the discussion towards the significance of using different media to draw general conclusions.

## Materials and methods

### Animals

Adult male (3–5 months) and female (1.5–5 months) B6CF1 hybrid (C57BL/6xBALB/c) and C57BL/6 mice were used. Animals were housed in the corresponding facility at Instituto de Biología y Medicina Experimental (IBYME) and were maintained with food and water ad libitum in a temperature controlled room (21–23 °C) with light:dark (12:12 h, lights on: 7:00 AM) cycle. Mice were humanely euthanized in the home cage by CO_2_ inhalation followed by cervical dislocation before gamete collection. Approval for the study protocol was obtained from the Institutional Animal Care and Use Committee of IBYME (No. 08/2021). Experiments were performed in accordance with the Guide for Care and Use of Laboratory Animals published by the National Institutes of Health (NIH). The study is reported in accordance with ARRIVE guidelines.

### Preparation of media

All the media were prepared with molecular grade reagents (Merck MilliPore (Burlington, MA), Sigma-Aldrich (St Louis, MO) and J.T. Baker (Phillipsburg, NJ)) dissolved in hexadistilled water (Braun, Buenos Aires, Argentina). Additionally, streptomycin-SO_4_ and penicillin G (Gibco, Grand Island, NY) were included in the formulation. Phenol red (ICN Biomedicals, Chillicothe, OH) was added as a pH indicator and, in the case of TYH containing HEPES (TYH (HEPES)), pH was adjusted to 7.3–7.4 with NaOH (Merck Millipore). Finally, media were filtered through 0.22 µm membranes and stored at 4 °C. At least three independent preparations of each medium were used along all the experimental procedures. Bovine serum albumin (BSA; cat. No.: A7906, Sigma-Aldrich) in the corresponding concentration (see Table 1) was added the day before the experiment, and complete media were then equilibrated overnight at 5% CO_2_ (v/v), 37 °C and saturated humidity, except for TYH (HEPES), which was equilibrated at 37 °C.

### Sperm collection and in vitro capacitation

To recover spermatozoa in the different media, each cauda epididymis of two males was dissected within a droplet of the respective medium (150 μl under paraffin oil (Ewe, Sanitas SA, Buenos Aires, Argentina)), letting sperm swim out for 10 min (fresh sperm). After this, aliquots were diluted in water and the number of sperm heads was recorded with a standard method using a Neubauer chamber under a light microscope (× 400). In addition, sperm viability was determined by dye exclusion using 0.5% (w/v) Eosin Y (Sigma) and the percentage of unstained cells was scored under a light microscope (× 400).

Aliquots of the “swim-out” suspensions were directly added to 300 μl of the matching capacitation medium under paraffin oil to give a final concentration of 5–7 × 10^6^ sperm/ml. It is important to note that the volume of “swim-out” required to obtain the final sperm concentration was higher for C57BL/6 than for B6CF1 samples. Sperm suspensions were then incubated for 90 min at 37 °C in an atmosphere with 5% (v/v) CO_2_ in air.

### Evaluation of sperm functional parameters

#### Phosphorylation of PKA substrates

PKA substrate phosphorylation was assessed as previously reported^37^. After capacitation, sperm suspensions were washed, resuspended in the Laemmli sample buffer^49^, boiled and centrifuged. Supernatants were boiled again in the presence of 70 mM 2-β-mercaptoethanol. Proteins (corresponding to 3 × 10^6^ sperm/lane) were separated by SDS-PAGE and transferred to a nitrocellulose membrane. After blocking with 2% (w/v) skim milk in PBS-Tween 0.1% (v/v), membranes were probed with anti-pPKAs antibody (1:1000; clone 9624, Cell Signaling Technology, Danvers, MA). Next, membranes were washed and incubated with peroxidase-conjugated secondary antibody (1:4000; Vector Laboratories, Burlingame, CA). The immunoreactive proteins were detected by ECL Western blotting kit (Thermo Fisher, Waltham, MA). Images were captured with G:BOX GENI (Syngene, Synoptics Ltd, Cambridge, England) according to the manufacturer’s instructions. Membranes were then stripped and probed with anti-β-tubulin antibody (1:3000; Sigma-Aldrich) as loading control. For quantification, ImageJ software (http://imagej.nih.gov/ij) was used, and the pixel intensity obtained for each lane was relativized to the pixel intensity of the loading control.

#### Computer-assisted sperm analysis

Motility was evaluated by CASA as previously described^19,50^. After capacitation, sperm aliquots (15 μl) were placed between pre-warmed slides and cover slips (22 × 22 mm) to create a chamber with 30 μm depth. The samples were then examined at 37 °C using the Sperm Class Analyzer^Ⓡ^ system (SCA v.6.2.0.1., Microptic SL, Barcelona, Spain). Drifting was set in 25 μm/s. At least 200 sperm distributed in a minimum of 10 different microscope fields were evaluated (30 frames acquired at 60 Hz for each measurement). The following parameters were assessed: curvilinear velocity (VCL, μm/s), straight line velocity (VSL, μm/s), average path velocity (VAP, μm/s), linearity (LIN,%), straightness (STR,%), oscillation index (WOB,%), amplitude of lateral head displacement (ALH, μm) and beat cross frequency (BCF, Hz). Sperm were considered hyperactivated when presenting VCL ≥ 271 μm/s, LIN < 50% and ALH ≥ 3.5 μm (Fig. 3a).

#### Acrosome reaction

The acrosomal status was evaluated in fresh and capacitated sperm exposed or not to progesterone (P4, 30 μM for B6CF1 and 40 μM for C57BL/6, (Sigma-Aldrich)) during the last 15 min of capacitation. Sperm suspensions were stained with Coomassie Brilliant Blue, as previously described^50^. Briefly, after fixation with 1 vol. of 8% (w/v) paraformaldehyde (Biopack, Buenos Aires, Argentina) in PBS, sperm were washed with 0.1 M ammonium acetate (Merck Millipore), pH: 9, mounted on slides, and air dried. Slides were washed by successive immersions in water, methanol (Biopack), and then incubated in 0.22% (w/v) Coomassie Brilliant Blue G250 (Bio Rad, Hercules, CA) solution (50% (v/v) methanol and 10% (v/v) acetic acid (Biopack)) for 10 min. After staining, slides were thoroughly washed with distilled water, mounted and immediately observed. Four hundred spermatozoa were evaluated in each treatment under a light microscope (× 400). Sperm were scored as acrosome-intact when a bright blue staining was observed in the dorsal region of the head or as acrosome-reacted when no labeling was observed (Fig. 5a).

### IVF and early embryo development assays

IVF assays were carried out as previously reported^40^. B6CF1 and C57BL/6 female mice were superovulated by injection of equine chorionic gonadotropin (eCG, 5 UI, Syntex, Buenos Aires, Argentina), followed by human chorionic gonadotropin (hCG; 5 UI, Syntex) 48 h later. Cumulus-oocyte complexes (COC) were collected from the oviducts 13–14 h after hCG administration and pooled. When needed, cumulus cells were removed by incubating the COC in 0.3 mg/ml hyaluronidase (type IV, Sigma-Aldrich) for 3–5 min, and ZP was dissolved by treating eggs with acid Tyrode’s solution (pH 2.5) for 10–20 s^51^. It is important to mention that egg collection and gamete co-incubation were performed in the matching capacitation medium.

Taking into consideration the diverse reproductive performances of the animal colonies, the conditions of the IVF assays were adjusted in each case. For B6CF1 gametes, COC were inseminated with capacitated sperm at a final concentration of 3–5 × 10^5^ sperm/ml and ZP-intact eggs with 5–7 × 10^5^ sperm/ml, and gametes were co-incubated for 2.5 h. In the case of C57BL/6 gametes, COC were inseminated with a final concentration of 5–7 × 10^5^ sperm/ml, and ZP-intact eggs with 7–9 × 10^5^ sperm/ml, and gametes were co-incubated for 3 h. In all cases, eggs were then transferred to the corresponding fresh medium, and the percentage of two-cell embryos was recorded the following morning (Fig. 6b). For gamete fusion assays, ZP-free eggs were inseminated with sperm capacitated in the different media (5–7 × 10^4^ sperm/ml), and gametes were co-incubated for 1 h. Eggs were then fixed with 2% (w/v) paraformaldehyde in PBS, stained with 10 μg/μl Hoechst 33342 (Sigma-Aldrich), mounted on slides, and analyzed under a Nikon Optiphot microscope (Nikon, Tokyo, Japan) equipped with epifluorescence optics (× 250). Eggs were considered fertilized when at least one decondensing sperm nucleus was observed in the cytoplasm.

Two-cell embryos obtained in IVF with COC were further incubated in KSOM (95.7 mM NaCl, 2.5 mM KCl, 1.7 mM CaCl_2_, 0.2 mM MgSO_4_, 0.4 mM KH_2_PO_4_, 25 mM NaHCO_3_, 0.2 mM glucose, 0.2 mM Na-pyruvate, 10 mM Na-lactate, 0.01 mM EDTA)^52^ supplemented with essential and non-essential amino acids (Gibco), 1 mM glutamine (Gibco) and 1 mg/ml BSA for 72 h to evaluate the development to blastocyst stage^46^.

All incubations were carried out in 50 µl droplets of each medium, under paraffin oil at 37 °C in an atmosphere of 5% (v/v) CO_2_ in air.

### Statistical analysis

Calculations were performed using the Prism 8.0 software (GraphPad Software, La Jolla, CA). Data was checked for normality (Shapiro–Wilk test) and homoscedasticity (Brown-Forsythe or Spearman’s tests depending on the number of compared factors). Comparisons were then made by one-way ANOVA (ANalysis of VAriance) for all the analyzed variables, except for the percentage of viability and acrosome-reacted sperm for which two-way ANOVA was used. In all cases, Fisher’s LSD post-test was applied. Data represent the mean ± SEM of independent experiments. Differences were considered significant when *p* < 0.05.

### Supplementary Information

Supplementary Information.

## Data Availability

All data generated or analyzed during this study are included in this published article.

## References

[1] Austin CR (1951). Observations on the penetration of the sperm in the mammalian egg. Aust. J. Sci. Res. B.

[2] Chang MC (1951). Fertilizing capacity of spermatozoa deposited into the fallopian tubes. Nature.

[3] Yanagimachi, R. Mammalian fertilization. In *The Physiology of Reproduction* (eds Knobil, E. & Neill, J. D.) 189–317 (1994).

[4] Toyoda Y, Yokoyama M, Hosi T (1971). Studies on the fertilization of mouse eggs in vitro. In vitro fertilization of eggs by fresh epididymal sperm. Jpn. J. Anim. Reprod..

[5] Gervasi MG, Visconti PE (2016). Chang’s meaning of capacitation: A molecular perspective. Mol. Reprod. Dev..

[6] Yanagimachi R (2022). Mysteries and unsolved problems of mammalian fertilization and related topics. Biol. Reprod..

[7] Aguilar J, Reyley M (2005). The uterine tubal fluid : Secretion, composition and biological effects. Anim. Reprod. Sci..

[8] Gardner DK, Lane M, Calderon I, Leeton J (1996). Environment of the preimplantation human embryo in vivo: Metabolite analysis of oviduct and uterine fluids and metabolism of cumulus cells. Fertil. Steril..

[9] Harris SE, Gopichandran N, Picton HM, Leese HJ, Orsi NM (2005). Nutrient concentrations in murine follicular fluid and the female reproductive tract. Theriogenology.

[10] Fraser LR, Drury LM (1975). The relationship between sperm concentration and fetilization in vitro of mouse eggs. Biol. Reprod..

[11] Nakagata N (1996). Use of cryopreservation techniques of embryos and spermatozoa for production of transgenic (Tg) mice and for maintenance of Tg mouse lines. Lab. Anim. Sci..

[12] Navarrete FA (2019). Transient sperm starvation improves the outcome of assisted reproductive technologies. Front. Cell Dev. Biol..

[13] Lamoreux ML, Delmas V, Larue L, Bennett DC (2010). The Colors of Mice.

[14] Gómez-Elías MD (2019). Association between high-fat diet feeding and male fertility in high reproductive performance mice. Sci. Rep..

[15] Krapf D (2010). Inhibition of Ser/Thr phosphatases induces capacitation-associated signaling in the presence of Src kinase inhibitors. J. Biol. Chem..

[16] Toyoda Y, Yokoyama M (2016). The early history of the TYH medium for in vitro fertilization of mouse ova. J. Mamm. Ova Res..

[17] Kito S (2004). Improved in vitro fertilization and development by use of modified human tubal fluid and applicability of pronucleate embryos for cryopreservation by rapid freezing in inbred mice. Comp. Med..

[18] Balbach M (2020). Metabolic changes in mouse sperm during capacitation. Biol. Reprod..

[19] Giaccagli MM (2021). Capacitation-induced mitochondrial activity is required for sperm fertilizing ability in mice by modulating hyperactivation. Front. Cell Dev. Biol..

[20] Luque GM (2021). Cdc42 localized in the CatSper signaling complex regulates cAMP-dependent pathways in mouse sperm. FASEB J..

[21] Lu Y (2023). 1700029I15Rik orchestrates the biosynthesis of acrosomal membrane proteins required for sperm–egg interaction. Proc. Natl. Acad. Sci..

[22] Wigger M (2023). Successful use of HTF as a basal fertilization medium during SEcuRe mouse in vitro fertilization. BMC Res. Notes.

[23] Chen Y (2000). Soluble adenylyl cyclase as an evolutionarily conserved bicarbonate sensor. Science (80-.).

[24] Baro Graf C (2020). Everything you ever wanted to know about PKA regulation and its involvement in mammalian sperm capacitation. Mol. Cell. Endocrinol..

[25] Suarez SS (2008). Control of hyperactivation in sperm. Hum. Reprod. Update.

[26] Balbach M, Beckert V, Hansen JN, Wachten D (2018). Shedding light on the role of cAMP in mammalian sperm physiology. Mol. Cell. Endocrinol..

[27] Nolan MA (2004). Sperm-specific protein kinase A catalytic subunit Cα2 orchestrates cAMP signaling for male fertility. Proc. Natl. Acad. Sci. U. S. A..

[28] Hess KC (2005). The “soluble” adenylyl cyclase in sperm mediates multiple signaling events required for fertilization. Dev. Cell.

[29] Branham MT, Mayorga LS, Tomes CN (2006). Calcium-induced acrosomal exocytosis requires cAMP acting through a protein kinase A-independent, Epac-mediated pathway. J. Biol. Chem..

[30] Wertheimer E (2013). Compartmentalization of distinct cAMP signaling pathways in mammalian sperm. J. Biol. Chem..

[31] Goodson SG (2012). Metabolic substrates exhibit differential effects on functional parameters of mouse sperm capacitation. Biol. Reprod..

[32] Balbach M (2023). Capacitation induces changes in metabolic pathways supporting motility of epididymal and ejaculated sperm. Front. Cell Dev. Biol..

[33] Schmidt CA (2024). Pyruvate modulation of redox potential controls mouse sperm motility. Dev. Cell.

[34] Ren D (2001). A sperm ion channel required for sperm motility and male fertility. Nature.

[35] Martínez-López P (2011). TRPM8 in mouse sperm detects temperature changes and may influence the acrosome reaction. J. Cell. Physiol..

[36] Romarowski A (2016). A specific transitory increase in intracellular calcium induced by progesterone promotes acrosomal Exocytosis in mouse sperm. Biol. Reprod..

[37] Weigel Muñoz M (2018). Influence of the genetic background on the reproductive phenotype of mice lacking Cysteine-RIch Secretory Protein 1 (CRISP1). Biol. Reprod..

[38] Fraser LR (1987). Minimum and maximum extracellular Ca2+ requirements during mouse sperm capacitation and fertilization in vitro. J. Reprod. Fertil..

[39] Visconti P (1995). Capacitation of mouse spermatozoa. I. Correlation between the capacitation state and protein tyrosine phosphorylation. Development.

[40] Da Ros VG (2008). Imparing sperm fertilizing ability in mice lacking cysteine-rich secretory protein 1 (CRISP 1). Dev. Biol..

[41] Miyata H (2015). Sperm calcineurin inhibition prevents mouse fertility with implications for male contraceptive. Science (80-.).

[42] Brukman NG (2016). Fertilization defects in sperm from Cysteine-rich secretory protein 2 (Crisp2) knockout mice: Implications for fertility disorders. Mol. Hum. Reprod..

[43] Carvajal G (2018). Impaired male fertility and abnormal epididymal epithelium differentiation in mice lacking CRISP1 and CRISP4. Sci. Rep..

[44] Shimada K, Kato H, Haruhiko M, Ikawa M (2019). Glycerol kinase 2 is essential for proper arrangement of crescent-like mitochondria to form the mitochondrial sheath during mouse spermatogenesis. J. Reprod. Dev..

[45] Miyata H (2021). SPATA33 localizes calcineurin to the mitochondria and regulates sperm motility in mice. Proc. Natl. Acad. Sci. U. S. A..

[46] Gómez-Elías MD, Munuce MJ, Bahamondes L, Cuasnicú PS, Cohen DJ (2016). In vitro and in vivo effects of ulipristal acetate on fertilization and early embryo development in mice. Hum. Reprod..

[47] Nakao S, Takeo T, Watanabe H, Kondoh G, Nakagata N (2020). Successful selection of mouse sperm with high viability and fertility using microfluidics chip cell sorter. Sci. Rep..

[48] Kito S, Ohta Y (2005). Medium effects on capacitation and sperm penetration through the zona pellucida in inbred BALB/c spermatozoa. Zygote.

[49] Laemmli UK (1970). Cleavage of structural proteins during the assembly of the head of bacteriophage T4. Nature.

[50] Curci L (2020). Functional redundancy and compensation: Deletion of multiple murine crisp genes reveals their essential role for male fertility. FASEB J..

[51] Nicolson GL, Yanagimachi R, Yanagimachi H (1975). Ultrastructural localization of lectin-binding sites on the zonae pellucide and plasma membranes of mamalian eggs. J. Cell Biol..

[52] Erbach GT, Lawitts JA, Papaioannou VE, Biggers JD (1994). Differential growth of the mouse preimplantation embryo in chemically defined media. Biol. Reprod..

