# Supplementary material for: A side-by-side comparison of different capacitation media in developing mouse sperm fertilizing ability
Source: Sci Rep. 2024 Jun 21;14:14287. doi: 10.1038/s41598-024-65134-w (PMC11192932; doi:10.1038/s41598-024-65134-w)
Supplement: Supplementary file 1 — Supplementary Information. [file 41598_2024_65134_MOESM1_ESM.pdf]

A Side-by-Side Comparison of Different Capacitation Media in Developing Mouse Sperm Fertilizing Ability

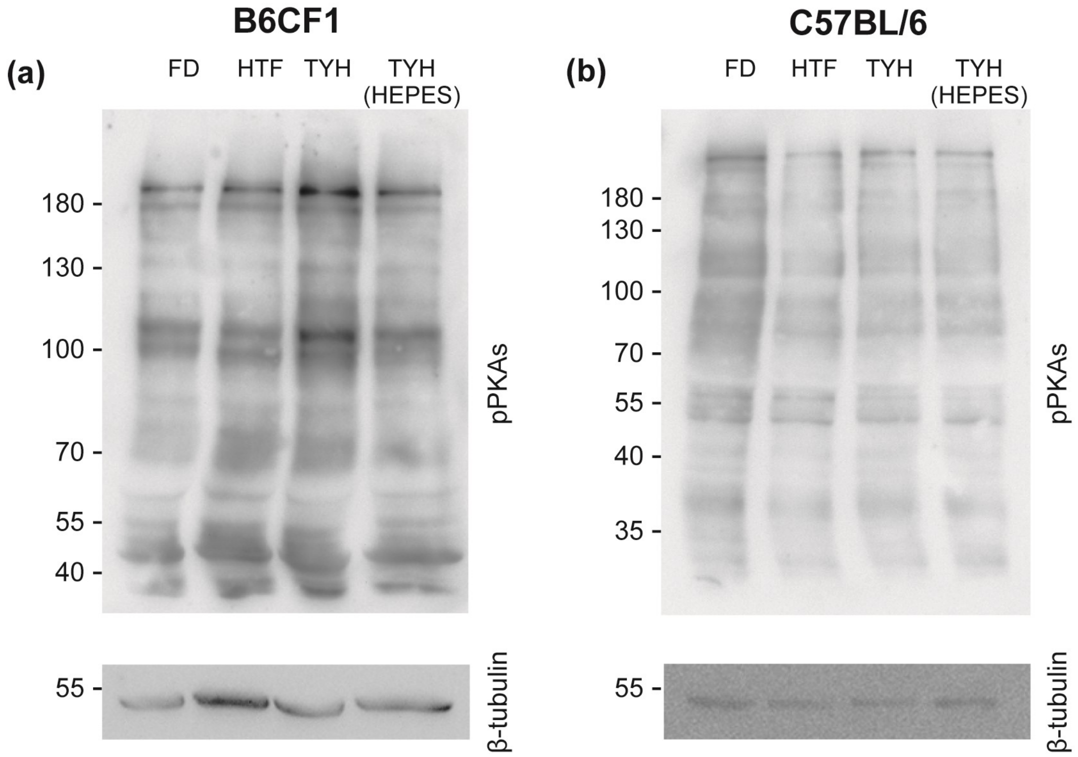

Supplementary Figure 1. Representative images of phosphorylation of PKA substrates (pPKAs) in the different media. Sperm from B6CF1 **(a)** and C57BL/6 **(b)** animals were capacitated in the corresponding media for 90 min. Levels of pPKAs were analyzed by Western blotting. β-tubulin was used as a loading control. Quantification of the band intensities of all the biological replicates is included in Figure 2.
